# Two-and-a-Half-Year Follow-Up Study with Freedom on Water through Stand-Up Paddling: Exploring Experiences in Blue Spaces and Their Long-Term Impact on Mental Well-Being

**DOI:** 10.3390/healthcare12101004

**Published:** 2024-05-13

**Authors:** Elisabeth Bomholt Østergaard, Pernille Wobeser Sparre, Jesper Dahlgaard

**Affiliations:** 1 Research Programme for Mind and Body in Mental Health, Research Center for Rehabilitation and Health Promotion, VIA University College of Aarhus, 8200 Aarhus, Denmark; pspa@via.dk (P.W.S.); jesd@via.dk (J.D.); 2 Blue Spirit Surf School, Ndr. Strandvej 42, 8400 Ebeltoft, Denmark

**Keywords:** blue health, blue space, blue nature, mental health, physical activity, stand-up paddling, SUP, long-term follow-up

## Abstract

Blue space interventions evidently have a positive impact on well-being and mental health, yet longitudinal studies on the lasting impact of such interventions are scarce. In this qualitative follow-up study with semi-structured interviews, we explored the long-term experiences over 18–42 months among six out of the initial eight women from the primary study, also including two instructors from the initial study. The participants, dealing with mental disorders, participated in the group-based intervention Freedom on Water, participating in stand-up paddling. Five main themes emerged from the empirical analysis: SUP as a catalyst for broadening horizons; learning: stepping out of the comfort zone; a break from diagnosis and rumination; connectedness to nature, specifically blue nature, and to the group; a life-changing journey; and a shift in mindset. The study revealed a long-term, life-changing impact of the program on participants’ well-being and mental health. Nature and blue space activities had become a greater part of their lives, improving their mental health with feelings of calmness, positivity, healing, and freedom. Stepping out of their comfort zone facilitated experiences of success and transformed their mindsets. Moreover, they experienced a break from rumination, and they became more outwardly focused, with confidence in themselves and their abilities, while making new friendships and engaging in new and different contexts.

## 1. Introduction

Throughout history, humans have sought out blue spaces—aquatic environments such as oceans, lakes, and rivers—for habitation, physical activity, relaxation, and healing [1]. Research shows that we are onto something when seeking water:

A systematic review from 2020 by Britton et al. emphasized that a growing body of evidence underpins the link between blue space interventions and increased psychosocial well-being [2]. In line with this evidence, a wave of blue space interventions for health and well-being has been launched. Several of these interventions involve physical activities conducted near, on, or in water, demonstrating positive impacts on the well-being and mental health of various target groups [2,3,4,5,6].

In a previous study [6] of a Danish project called Freedom on Water, combining blue space and physical activity, the findings revealed that participants experienced positive effects on their well-being and mental health. Self-confidence, a positive attitude towards oneself, intrinsic motivation, and social integration were increased, and rumination was decreased. While the short-term effects were significant, the question of whether the participants’ experiences could have a lasting influence on their well-being and mental health remained unanswered [6]. The study indicated the need for additional research to evaluate the long-term impacts on these domains.

A systematic review from 2020 highlighted a lack of longitudinal studies and concluded that due to this shortage, whether the benefits associated with participation in blue space interventions are sustained and whether the benefits could vary across the life course, remain untested [2].

A scoping review of the qualitative and quantitative research evidence on surf therapy from 2020 concluded that future research is needed to analyze the long-term impact and sustainability of surf therapy programs and to gain a better understanding of the appropriate time frame, quantity, and quality of interventions for sustaining long-term improvements in physical and psychosocial health and wellbeing [3].

In an era where mental health is on a downward spiral and mental health disorders are on upward spiral [7,8,9], it is necessary to explore new initiatives and supplements to existing treatments [7,8,9]. There are no quick fixes for mental disorders, and therefore, it becomes of great importance to investigate the long-term effects of blue space interventions, to examine causality, and to inform public health interventions.

The aim of this follow-up study was to explore the long-term experiences of people with mental disorders participating in Freedom on Water, including stand-up paddling (SUP), and to explore whether and how these experiences influenced their well-being and mental health in the long run. Furthermore, the aim was to explore the experiences of the instructors and to generate valuable insights for health professionals in the field of blue space interventions for mental health.

### 1.1. Definition of Freedom on Water

Surf & SUP Denmark [10] developed the project Freedom on Water (translated from Danish “Fri på vandet”) [11], and it was started in 2020. The project offered surfing and stand-up paddling (SUP) for adults living with a mental disorder such as depression, anxiety, or stress. The project was financially supported by the Danish Outdoor Council [12] in cooperation with ten associations across Denmark, where the activities took place and were carried out by volunteer instructors. The purpose of the project was to focus on being present in the moment and experiencing nature, thereby giving the participants a break from the challenges in their everyday lives.

### 1.2. Definition of Mental Health

The study uses the WHO’s definition of mental health as a state of mental well-being that enables people to cope with the stresses of life, realize their abilities, learn well and work well, and contribute to their community. It is an integral component of health and well-being that underpins our individual and collective abilities to make decisions, build relationships, and shape the world we live in. Mental health is a basic human right. And it is crucial to personal, community, and socio-economic development [13]. Mental health is more than the absence of mental disorders. It exists on a complex continuum, which is experienced differently from one person to the next, with varying degrees of difficulty and distress and potentially very different social and clinical outcomes [13]. Mental health conditions include mental disorders and psychosocial disabilities, as well as other mental states associated with significant distress, impairment in functioning, or risk of self-harm. People with mental health conditions are more likely to experience lower levels of mental well-being, but this is not always or necessarily the case [13].

## 2. Materials and Methods

### 2.1. Participants

Eight participants from the original group of ten included in our study conducted in 2021, published in 2023 [6], participated in this follow-up. These eight participants consisted of six women, aged 18–60, living with one or more mental disorders such as PTSD, anxiety, stress, schizophrenia, etc. (P1, P2 …P6) (primary participants), along with two female instructors (I1, I2) (secondary participants), and they were all a part of the Freedom on Water project [11], conducted at ten different locations across Denmark.

Our inclusion criteria in the study from 2021, published in 2023 [6], were men and women of any age with mental disorders such as PTSD, anxiety, stress, schizophrenia, etc., participating in the Freedom on Water project, as well as instructors from the same project. We aimed to recruit both men and women; however, no men responded, and we chose not to focus on age in our study because we were interested in the phenomenon and in people’s experiences.

Our inclusion criteria in this two-and-a-half-year follow-up study were participation in the project Freedom on Water, beginning two and a half years ago.

### 2.2. Design

In our initial study from 2021, published in 2023 [6], we conducted qualitative anthropological fieldwork [14], with participant observation [15] and different types of qualitative interviews, on the water as well as on the ground, for three months in February, April, and May of 2021 in Denmark.

After two and a half years, we conducted the present follow-up study, employing semi-structured interviews in August and September 2023 with six of the eight women from the initial study and with the two instructors, also from the same initial study. The design and approach were qualitative. We conducted a qualitative descriptive study [16] using anthropological fieldwork [14], using semi-structured interviews with exploratory research aims and research questions to explore and understand the participants’ experiences.

Four participants were still participating in one SUP session per week in the club from April through October, with a break in the wintertime. Participation could vary depending on mental state and other life events. The sessions typically lasted two hours, and some of them were followed up by social initiatives. Another two participants chose to pursue new activities one and a half years after the intervention.

A typical SUP session consisted of an introduction to the session, changing into wetsuits and getting the equipment ready, paddling on the water (with distances varying, depending on the weather and the participants’ energy), mindfulness on the water, getting back on land to change and pack up, final gathering, and social chatting, either outside or inside the club house. The participants did learn basic SUP skills at their own pace, but in general, the focus of the sessions was to have a free space in nature and not to teach specific technical skills, etc.

The two instructors who participated in this study were both certified SUP instructors with valid first aid certificates. Before the intervention started, Surf & SUP Denmark facilitated a two-hour workshop regarding working with people with mental disorders, which was taught by a psychologist. It was also possible for the instructors to continuously contact the psychologist for coaching, if needed.

Author Pernille Wobeser Sparre (P.W.S.), who conducted the fieldwork in 2021, also carried out the interviews in the follow-up study in 2023. She, therefore, had already built a relationship with the participants.

The reason for using a qualitative research design was to explore the experiences of people with mental disorders participating in SUP and to explore if and how the experiences had a long-term impact on their well-being and mental health. Our perspective was phenomenological to explore and understand the participants’ lifeworld [17].

The theoretical analytical perspectives were selected after finishing the empirical data collection and analysis in order to emphasize the importance of the empirical data itself and to highlight the fact that the empirical data was superior to the theory, as well as to avoid letting the theoretical perspectives overrule the empirical data, as underlined by, e.g., Wolcott, Bundgaard and Mogensen, and others [18,19,20,21].

The semi-structured interviews with primary and secondary participants were based on interview guides (Appendix A.1 and Appendix A.2). The instructors also met with the participants during the wintertime when they were not on the water, and both instructors had been involved in the project since its beginning. This contributed to enriching our understanding of the project from an alternate perspective. The semi-structured interviews took place at locations known and chosen by the participants, and were conducted either face-to-face, at a coastal area location, or on the telephone.

**Figure 1:**
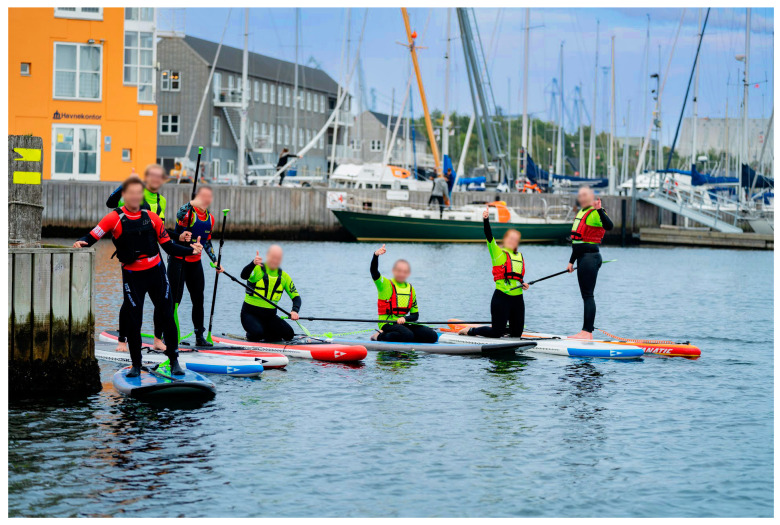
SUP activity, used with permission from Surf & SUP Denmark (Photo: Jakob Gjerluff, Gjerluff Photography).

Figure 1 presents a photo from another context (to protect the participants’ anonymity and integrity) to illustrate the SUP activity.

### 2.3. Preunderstanding

By explicating preunderstanding, the ability to release yourself from the preunderstanding is increased [22]. We liberated ourselves from preconceived notions or preunderstandings by clarifying them and writing them down.

Our preunderstanding was affected by the results from the initial study, i.e., experiences of mental health being affected positively by participating in SUP, with increased self-confidence, skills, forgetting oneself, positive attitude towards oneself, intrinsic motivation, and social integration, along with decreased rumination. Under full concentration, while managing the (bodily) challenges on the SUP board, the participants’ rumination diminished or disappeared, and they felt present in the moment. We made ourselves open-minded towards the potential long-term impacts of this program on mental health.

### 2.4. Entering the Field

To recruit participants author P.W.S. reached out to the head instructor of Freedom on Water. It was the same head instructor who made entering the field possible in the initial study. A letter with information regarding the study was distributed to the participants through the instructor. The participants either replied directly to author P.W.S. or to the head instructor if they wanted to participate in the study.

### 2.5. Ethics

In a holistic mind–body approach, there are potentially unexplored benefits for people with mental disorders combining suitable challenging (bodily) activities, the outdoors, blue nature, and social activities in order to positively influence mental health. However, the long-term experiences and benefits of participating in water-based activities, including stand-up paddleboarding (SUP), for individuals facing mental challenges and disorders have not been thoroughly explored, emphasizing the significance of this study.

The study was conducted in accordance with The Declaration of Helsinki [23]. Our study received written approval from The Central Denmark Region Committee on Health Research Ethics, Skottenborg 26, DK-8800 Viborg, Denmark, on 7 April 2021 (record number: 1-10-72-1-21, query 79). Informed consent was obtained from all participants involved in the study. All were written and verbally informed of the goals and reasons for doing the research, along with the methods; that participation was voluntary, that they could withdraw from the study at any time, and that our professional confidentiality and their complete anonymity were ensured. Written informed consent was obtained from all participants to publish this paper.

When initiating the interviews, author P.W.S. introduced herself, provided a detailed explanation of the study’s objectives, discussed the execution of the fieldwork, and encouraged questions. This approach ensured that the participants were thoroughly informed.

We anonymized the data and names of the participants to protect individual confidentiality [23,24]. The participants’ names were anonymized with the following pseudonyms: P1, P2, P3, P4, P5, P6, I1, and I2. The project Freedom on Water, developed by Surf & SUP Denmark, is unique and the only one of this kind in Denmark; consequently, it is impossible to anonymize this program. However, the project is implemented at ten locations all over the country, and the precise location of our study is anonymous; therefore, this does not affect the protection and anonymity of the participants. Denmark has an abundance of water, including the sea and seaside around the country, with many lakes and rivers; thus, we use the designation “water” instead of lake, sea, and river to anonymize the precise locality in Denmark and to protect the participants’ identity.

We refer to participants as “people with a mental disorder”, using people-first language [25,26,27,28]. The aim is to emphasize a holistic perspective to signal the superior importance of the person rather than the disease, by describing that the person has a disease, and not is the disease [24].

If the study potentially affected the participants negatively, we were prepared for a dialogue regarding this effect. On the contrary, the study could also affect the participants positively, i.e., by providing: more conscious about what is good for me, what gives me calm, a feeling of being present in the moment, a break from rumination, courage to move out among other people, energy, the capability of changing my lifestyle, etc.

The researcher’s position potentially influences the participants’ answers in different ways, whether the researcher is, e.g., a psychologist, doctor, social worker, or physiotherapist, and this potentially influences the researchers’ interest, along with the potential interview questions, because of the different areas of expertise. From the outset, P.W.S. portrayed herself as researcher and was mindful of not assuming the role of physiotherapist to prevent responses primarily centered on the body and physical activity. However, over the course of the research period and as confidence grew among participants, information was more openly shared. The fact that the researcher was a physiotherapist was no longer kept confidential when participants inquired about the background of P.W.S.

Author P.W.S. is familiar with the context of SUP. She has enjoyed SUP several times herself, she has experienced great pleasure being on the water, getting an adrenaline rush by feeling the power of water and nature, and it made her feel like she was part of something much bigger, in which the worries of everyday life diminished. In this way, author P.W.S. became a “native”. Sometimes, it is questioned whether it is possible for the “native” anthropologist to create a necessary distance from the participants and the field, but on the other hand, it is also assumed that the “native” anthropologist is able to contribute with more authentic knowledge than the not-native anthropologist [29].

There were no affiliations/relationships between the participants and the researchers.

This research received no external funding.

### 2.6. Data Collection

Semi-structured interviews were recorded using an iPhone, audio only, and were transcribed in full length, including pauses and repetitions. During transcription, persons, places, and diseases were blurred to protect anonymity [23,30]. 

During all interviews, observations concerning body language and nonverbal communication were noted as valuable markers, contributing knowledge that could either strengthen or weaken the statements. The transcribed texts were read simultaneously while listening to the recordings to ensure the reproduction and preservation of the original meanings [30]. Reading the transcriptions caused a reliving of the interview, resulting in new reflections and perspectives supplying the basis of the data analysis [22,31].

### 2.7. Thematic Analysis of the Empirical Data

The empirical data were analyzed using thematic analysis following Malterud’s four-step approach, based on Giorgi’s psychological phenomenological thematic analysis [31].

In the first step, we thoroughly reviewed the transcribed empirical material, formed initial impressions, and identified the following preliminary themes: gateway experiences; like-minded people; learning: stepping out of the comfort zone; nature and water; a break for the brain; and a life changing journey.

In the second step, we identified empirical data for each theme (example in Table 1).

In step three, we condensed the empirical data for each theme to get the essence of each theme. We expressed the essences using synthetic quotations consisting of several compiled quotations and observations with equal meaning (example in Table 2).

In step four, each theme was re-contextualized to an academic text. The final themes are the headings in Section 3.

### 2.8. Analytical Perspectives

After the empirical analysis, we discussed our results with perspectives from Barbara Fredrickson [32], Albert Bandura [33], Deci and Ryan [34,35], Mihaly Csikszentmihalyi [36], Anne Kirketerp [37], Guadagnoli and Lee [38], Maurice Merleau-Ponty [39], Wilson and Kellert [40,41], and Gert Biesta [42]. We also discussed our results in relation to studies from the area of blue space interventions: Benninger et al. [3], Britton et al. [2], Capurso and Borsci [43], Grocott and Hunter [44], Hayhurst et al. [45], McCulloch et al. [46], Ritchie et al. [47], Wilkie et al. [48], Killingsworth and Gilbert [49], Pearce et al. [50], Pasanen et al. [51], Djernis et al., Capaldi et al. [52], and Richardson and Butler [53].

## 3. Results

### 3.1. Themes

Five main themes were identified from the data: (1) SUP as a catalyst for the broadening of horizons; (2) learning: a step out of the comfort zone; (3) a break from diagnosis and rumination; (4) connectedness and being part of something bigger; (4a) connectedness to nature, specifically blue nature; (4b) connectedness to the group of peers; (5) a life changing journey; a mindset shift.

#### 3.1.1. Theme 1: SUP as a Catalyst for the Broadening of Horizons

Experiences on the SUP board served as a catalyst for participants to embark on new activities. In recent years, several participants had initiated new activities. For instance, one participant had recently begun participating in a group-based nature program, and in connection with this, she described:

P2: “I didn’t really know if I could, and now I’m almost like the volunteer organizer of it all. It has just changed so much for me, and it is Freedom on Water that started it all, it’s that 100% yes. I began to learn a bit about my strengths even though I was actually very miserable at the time”.

In general, participants explained that their experiences with Freedom on Water had helped them realize their inner resources and enabled them to engage socially in a group. These experiences also boosted their self-confidence and collectively gave them the courage to broaden their horizons and explore and implement new activities in their everyday lives.

Three participants (P4, P5, P6) had begun practicing SUP outside of the Freedom on Water sessions by going on their own or participating in “club evenings”, which are social SUP events organized by the SUP club for all members. In both scenarios, the participants mentioned that when they initially joined Freedom on Water, they were hesitant to paddle alone or with the “regular” club members. However, thanks to the positive experiences gained from Freedom on Water, they have gained more confidence, which has expanded their willingness to venture out onto the water in various settings.

For other participants, their experiences had opened them up to new opportunities which did not include SUP. P3 expressed that Freedom on Water had opened so many doors for her. She had now joined the cinema club, she had made new friends, and she had started to meditate. The mindfulness element in Freedom on Water inspired her to start meditating because she realized that she experienced a benefit from it.

Overall, the participants had embraced a range of new activities, spanning from social gatherings to water gymnastics, discovering novel ways to appreciate nature, among other pursuits.

#### 3.1.2. Theme 2: Learning: A Step out of the Comfort Zone

Stepping out of one’s comfort zone fostered growth and learning, which the participants experienced in various ways. Some participants shared their reflections:

P1: “I remember once we stood on the board doing some yoga, and then I fell into the water, and that feeling, it’s one of those good experiences. Well, I don’t think anyone even noticed I fell into the water because I got up so quickly. But just the success of coming home and thinking, ‘Wow, I handled that really well, yes!’”

P4: “Challenging, fun, boundary-pushing, but also instructive because you really get exposed to a lot of things—because when you fall in the water, you’re very vulnerable, and, um, I also think you’ve learned a lot about yourself, what you can do, what you can’t do, trusting yourself”. 

P4: “To listen to my own instincts. The fact that I am better than I am telling myself. I don’t know how to explain it. Like there are more resources than there are in daily life because you get on the board, you are able to paddle, you are also able to be social, but also to gradually push your limits gradually, that it is not dangerous when you get scared on the water”.

Learning SUP as an activity had not been the only challenge for the participants. The participants also described the social aspect of their experiences as challenging. For some, it remained a challenge when new people joined the group. For example, P1 experienced that a period with new and (too) many people made it difficult for the instructors to keep an eye on all participants and negatively influenced the quality, while others have experienced a shift. 

P4: “Earlier it was impossible for me to let other people give me a hug, but gradually it has become okay for me to let the instructors and people in the club give me a hug, so, this is a huge victory”. Another participant reflected on her learning: P6: “So I’ve probably learned to be more open about my disease…. it has been for me…. yes, it is a big plus”.

Several participants (P3, P4, P5, P6) spoke positively of the instructors and of how their support also contributed to increase the participants’ confidence. A participant said: P5: “The instructor she is super good at boosting our self-confidence and stuff like that—that we can do it and that we can actually do more than we think”.

These changes are supported by the observations from the two instructors of the project, who described how they had experienced the participants’ growth. They observed that, gradually over time, the process of overcoming fears, combined with experiences of success, had led to an increase in confidence. During the past three and a half years, the instructors had observed how the participants had become more courageous and willing to step out of their comfort zones.

I1: “So there is absolutely no doubt that it has helped giving them a boost of self-confidence and security to push some boundaries they didn’t believe they could push, and for some I think it has also meant something that this has nothing to do with treatment, neither psychiatry nor the municipality. This is one’s own little haven where you can be as normal as possible, if you understand, you are not being looked upon as depressed, you are seen as a human being … and not as a disease or a citizen who costs money…”

The participants’ experiences encompassed various dimensions of learning. Apart from learning SUP as an activity, they have also gained insights into their own resources, enhanced their social development, and acquired the ability to step out of their comfort zones.

#### 3.1.3. Theme 3: A Break from Diagnosis and Rumination

The participants experienced a break from their rumination through physical activity, which included focusing on technique and balance, all while immersed in the sensory impressions of the surrounding nature. A participant reflected on the break she experienced on the water:

P5: “Yeah, it’s like, um, actually when I get out and sort of, when I am out, all those things that are swirling around in one’s head, and those things that are hard to handle, they’re not at the forefront. What fills me when I’m out, it’s just the freedom and feeling that you’re not bound by anything, and this community that we have in the team, that means a lot”.

Several participants (P3, P4, P5, P6) described how the calming effect they experienced on the water lasted throughout the rest of the day when they returned to land, leaving them feeling more relaxed and energized.

One participant also described how being on the water provided her with a break from her mental health condition, as she did not hear voices when she was on the water.

In general, the participants conveyed that the break they experienced on the water brought them a sense of calmness not attainable through any other activities in their daily lives.

When asked if other things or experiences could provide the same break from rumination, P3 answered: “Not in the same way, because I must concentrate to paddle and to avoid falling into the water, and I have to concentrate on what the other participants say … I listen to music at home to get a break, but this (SUP) is much better and gives me much more, and I am pleased with myself when I get home”.

#### 3.1.4. Theme 4: Connectedness and Being Part of Something Bigger

##### Theme 4a: Connectedness to Nature, Specifically Blue Nature

Through their experiences with Freedom on Water, the participants had realized that nature and water had a positive and calming impact on them. In general, they associated nature and water with feelings of calmness, positivity, a free space, well, and a sense of freedom. This newfound understanding motivated some participants to begin dedicating time to nature as a means of improving their mental health and overall well-being. A participant explained:

P4: “Those days where I’m really struggling with my disease, I go out and feel the nature as a calm and peaceful place and I just listen to the birds. I’ve bought my own SUP-board so when it is really bad, I can just go on the water on my own and get a free space there. So, nature has more become a free space rather than just a matter of course”. 

P3 expressed that she had experienced a hard childhood, and she remembered that walking along the beach, listening to the sound of the waves, gave her a free space and a space for positive thinking.

P2 responded that nature gave a movement in itself, a movement she did not need to be an active part of, a movement telling her: “Everything will be all right, everything is floating, you don’t need to do anything—this is what all this water has given me most of all”.

When going out in nature, P4 explained: “In some way I experience grounding, because I know where I am in the world, I can smell the rain and the forest floor, well it diverts the thoughts and makes me capable of directing my attention to other things”.

The interviews also revealed a change in the way the participants perceived nature. P1 described: “So I’ve never been crazy about water, and now I think it is amazing (…)” Another participant, P2, described: “(…) Now I’m like discovering nature every time I’m out for a walk then I see something new and interesting”. The participants expressed how they now had a greater appreciation for nature and emphasized that they had become more aware of the nature around them.

When recounting their experiences, the participants also emphasized that being on the water provided a distinct and unique perspective on spending time in nature, in contrast to being on land, and P2 mentioned that being in the water felt healing to her.

In general, the participants found it challenging to express in words how and why they felt benefits from being in, on, or by water; however, over time, it had become easier for them to express these feelings in words, compared their abilities during the initial study.

##### Theme 4b: Connectedness to the Group of Peers

Meeting and spending time together with like-minded people through Freedom on Water had led to new friendships, increased confidence in social skills, and a greater acceptance of their life situation. A participant emphasized:

P5: “So for me it has meant a lot to get out and be together with someone who were like-minded, like someone you don’t have to be accountable to and things like that, which has given me more courage to get out among people. It was a bit of my barrier; it was other people”.

In this way, Freedom on Water had worked as a catalyst for the evolution of the participants’ social lives, including other social interactions besides those provided by Freedom on Water.

Some of the participants (P3, P4, P5, P6) also described that the sense of belonging they felt through Freedom on Water was one of the reasons why they were still participating three and a half years after the project started. One participant said:

P3: “You feel that you’re a part of something big, bigger than I could create myself, like being a part of a workspace or a football team, you are a part of something. Well, when we actually don’t have a job, us four girlfriends call each other ‘colleagues’, because we don’t have colleagues and we miss them. To be part of something and to feel a sense of belonging to others that means a lot, instead of being labelled as ‘not capable’ (i.e., diagnosed with having a mental condition) as we otherwise have been needing to, in order to get incapacity benefit”. 

P3 added that meeting like-minded people had led to a better acceptance of her situation.

In general, it was meaningful for the participants to meet peers who understood their situation without the need to explain everything.

The two instructors also observed the participants’ social growth. They both noted that several participants had developed friendships. Those who had been involved for multiple years had become more at ease in the setting and within the group. The instructors had witnessed some participants becoming more open, helpful, and even taking leadership roles when new members joined the group, and P2 also explained that she took leadership in a new nature-intervention of which she had become a part.

It was highlighted that Freedom on Water offered the participants a constellation which included acceptance, support, and consideration, both among each other and the instructors.

#### 3.1.5. Theme 5: A Life Changing Journey; a Mindset Shift

P2: “I couldn’t have done without it. Otherwise, I don’t think I would be here today, if it had not been for Freedom on Water, I simply don’t think so (…) It was almost lifesaving for me. At the time I started, I couldn’t do anything”.

The participant went on to explain that by learning to shift her focus and be present in her body, she now, three and a half years later, believed she had an amazing life, despite her ongoing limitations. She had come to accept this reality instead of viewing it negatively.

Over time, Freedom on Water had brought about various changes in the lives of the participants. The participants emphasized a greater encouragement to become more involved in new activities, encompassing both social interactions and nature experiences. They had also gained insights into themselves through this process. P4 also shared with author P.W.S. that her overall mood had improved, and whenever she experienced a downturn, it was not as severe as it used to be.

Freedom on Water had had a positive impact on the participants’ daily lives. A participant said:

P3: “It has made me quickly get out of the house once a week regularly. It has helped me settle in faster and I’ve formed good friendships. It has made me always look forward to Thursday, um, it has overall helped me settle in town, both with friends, nature experiences, and accepting who I am. And yes, it has also helped me accept the fact that I am on early retirement which is something I used to be ashamed of and didn’t tell many people about. But today I can talk about it more easily and about that this is how my life is right now”.

Another participant reflected on her daily life, in which SUP was now playing a big part: P6: “So I was also feeling insecure about the fact that I was going to be together with other people in that way and with so many people—well, I think that has changed now. So, I’ve become happier, and I dare to call around so we can go on the water together (…)”.

The participants expressed that they had experienced benefits from participating.

**Figure 2:**
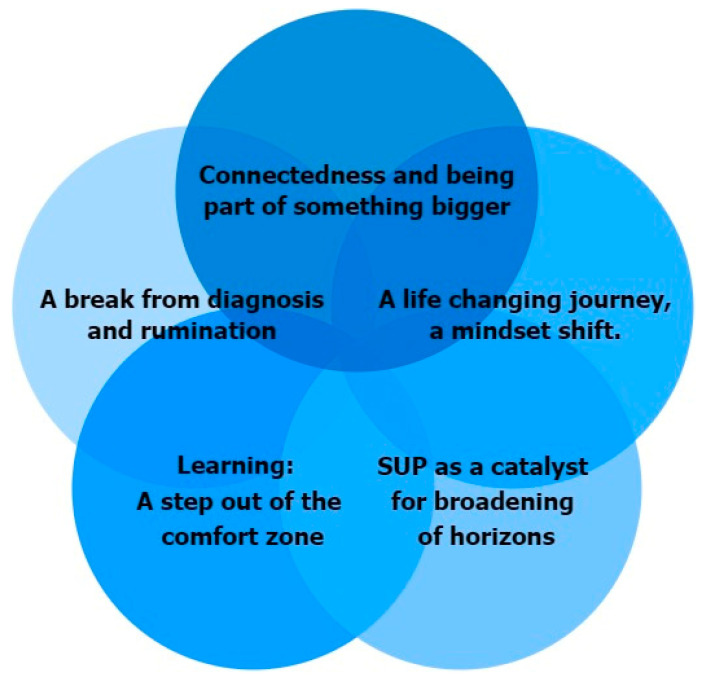
All themes overlapped each other, and together, they formed a holistic picture of the participants’ experiences regarding Freedom on Water.

In summary, the above-mentioned themes overlapped and worked in interaction with each other, and together, they created a holistic picture of the participants’ experiences, as Figure 2 illustrates. The themes had been experienced, to various extents, by all of the participants.

## 4. Discussion

The aim of this follow-up study was to explore the long-term experiences from people with mental disorders participating in Freedom on Water with SUP in a group of peers and to explore if and how the experiences had an impact on their well-being and mental health in the long run. Furthermore, the aim was to explore the experiences from the point of view of the instructors, and together, to create useful knowledge for health professionals in the area of blue space interventions for mental health.

Our main findings were that participating in Freedom on Water for a minimum of one and a half years up to three and a half years had a long-term life-changing impact on the participants’ mental and social life. It caused the participants to become capable of changing their lives by being more outwardly focused on society, with more confidence in themselves and their own abilities. They learned that stepping out of their comfort zones gave them experiences of success and pushed their boundaries. They experienced breaks from rumination that lasted longer than the SUP sessions, providing them with additional energy in their everyday lives. They achieved a feeling of being much more than just their diagnosis. They felt a colleagueship with the like-minded people in their group of peers, they made new friendships, and they began participating in different social contexts, some of them even taking on leadership roles. Freedom on Water had opened them up for and encouraged their participation in new activities on their own initiatives, and the participants had continued accessing these new opportunities.

Our findings are important because they fulfill the need for long-term research in the area of blue space interventions. Very few studies have considered the long-term effects of blue space interventions for mental health and mental well-being, and most of the existing studies include very short follow-up periods: two weeks, five months, 10 months, and in one study, one year [2,3,43,44,45,46,47,48]. No research, apart from our study, has considered the long-term effects after more than one year.

This discussion prompts the question: How can it be such a life changing journey, as for example one of the participants said, “I couldn’t have done without it?”.

Helping to understand the how, we sought help from the perspectives of Barbara Fredrickson [32], Albert Bandura [33], Deci and Ryan [34,35], Mihaly Csikszentmihalyi [36], Anne Kirketerp [37], Guadagnoli and Lee [38], Maurice Merleau-Ponty [39], Wilson and Kellert [40,41], and Gert Biesta [42]. We also discussed our results, referencing studies from the area of blue space interventions, conducted by Benninger et al. [3], Britton et al. [2], Capurso and Borsci [43], Grocott and Hunter [44], Hayhurst et al. [45], McCulloch et al. [46], Ritchie et al. [47], Wilkie et al. [48], Killingsworth and Gilbert [49], Pearce et al. [50], Pasanen et al. [51], Djernis et al., Capaldi et al. [52], and Richardson and Butler [53].

### 4.1. Theme 1: SUP as a Catalyst for the Broadening of Horizons

The participants highlighted that through their positive experiences with participating in SUP, they gained more confidence, which expanded their willingness and courage to venture out onto the water in various settings.

The participants learned about their strengths and inner resources, and participating in Freedom on Water functioned as a catalyst for perception and transformation by providing gateway experiences, thus broadening the participants’ horizons in several different ways. Overall, the participants had embraced a range of new activities, ranging from social gatherings to water gymnastics, discovering novel ways to appreciate nature, among other pursuits.

These findings are supported by Barbara Fredrickson’s broaden-and-build theory [32]. According to this theory, positive experiences and emotions build up personal resources and fuel individual differences in regards to resilience and the power of self-confidence. The positive experiences and emotions broaden horizons and facilitate personal growth. The theory also makes the bolder prediction that experiences of positive emotions, over time, build, and not just reflect, psychological resilience. From this perspective, positive emotions broaden the scopes of attention and cognition, enabling flexible and creative thinking and also increasing peoples’ enduring coping resources [32].

Another factor noted for gaining gateway experiences and the broadening of horizons was the importance of support. It was highlighted that Freedom on Water offered a constellation which included acceptance, support, and consideration, both from each other and from the instructors. The instructors supported them to feel safe, overcome fear, increase confidence, and they supported the ability to step out of the comfort zone and experience success. As the participants got to know and felt comfortable with each other, they also received the needed support from each other and no longer only from the instructors. The social aspect contributed to facilitate the participants’ experiences by creating a framework in which they received the courage to get out and learn new skills and develop competencies.

Bandura’s social cognitive theory confirms the importance of support from others and in this case, from the instructors and the other club members acting as role models [33]. According to this theory, support and recognition from the social surroundings are important factors for feeling safe and achieving the desired goals. People learn through observing, imitating, and modeling others’ behaviors. Other people can serve as role models, and observing others successfully participating in the activity can contribute to strengthen one’s self-efficacy. The role of the professional is important to ensure that the activity matches the participant’s level, being neither too easy nor too complicated.

According to the theories of Deci and Ryan regarding human needs and the self-determination of behavior [34], the participants’ experiences of relatedness, support, and acceptance were important factors for creating a foundation for a change of behavior, leading to increased autonomy. The social aspect of the participants’ experience was in line with the fulfilment of the psychological need relatedness, which can lead to increased well-being and social health, according to Deci and Ryan [35].

### 4.2. Theme 2: Learning: Stepping out of the Comfort Zone

For many participants, moving out into the water on an SUP board, being unfamiliar with SUP and accompanied by unknown people, involves stepping out of their comfort zones. The participants experienced stepping out of their comfort zones by participating in challenging activities that fostered growth and learning. As one of the participants described, she was afraid of falling into the water, when it suddenly happened. She fell into the water, and it simply turned out to be an experience of success because she managed to get up again so quickly that nobody noticed. She had the feeling of, “Wow, I did it well, yes!” (P1).

How can we understand the acquired courage to step out of one’s comfort zone, participating in challenging activities that foster growth, learning, and experiences of success?

According to the theories of Bandura and Deci and Ryan, support from others is certainly important for feeling safe, as a secure basis for daring to step out of one’s comfort zone and thereby, for achieving experiences of success [33,34,35].

Next, participating in activities that are neither too easy nor too challenging, but rather exactly as difficult and challenging as you need them to be in order to have to fully concentrate, i.e., with a balance between challenges and skills, then, according to the theories of Csikszentmihalyi, you have an optimal basis for receiving optimal experiences, sensing flow, and forgetting oneself [36]. Also according to the theories of Bandura, the balance between challenges and skills is important for gaining self-efficacy [33]. Activities which are neither too easy nor too challenging are the basis for learning and for stepping out of the comfort zone, leading to increased experiences of success and thereby, increased self-efficacy [33]. Guadagnoli and Lee even emphasize that participating in activities that are difficult enough that you fail a little, but not too much, offer the best potential for learning. This is described as the optimal challenge point [38].

When we venture into something new and unknown, then according to Biesta’s theories of world-centered education [42], we “have to open up and allow ourselves to be affected” [42] (p. 93). This experience of “not knowing” is connected with uncertainty and also with a risk, and precisely by “actively exposing ourselves to the new and unknown, we are exposed to the unknown (passive) and therefore we are vulnerable” [42] (pp. 93–94). Paradoxically, this vulnerability and passivity enables vigor, agency, and adequate responding. An inherent human intention to move and an experience of meeting the unknown enables our capacity to be influenced and affected, providing for changes, development, cognition, and learning [42].

### 4.3. Theme 3: A Break from Diagnosis and Rumination

In general, the participants experienced a break from their rumination and their diagnosis through participating in SUP, and the break they experienced on the water brought them a sense of calmness not attainable through any other activities in their daily lives.

The balance between skills and challenges and thereby, the difficulty of the activity, made the participants fully focused on the SUP activity and automatically brought their awareness to the present moment instead of allowing them to think about the past and the future, etc.

This is supported by the theories of Csikszentmihalyi, pointing out that through activities neither too easy nor too challenging, but exactly so difficult and challenging that full concentration is required, i.e., with a balance between challenges and skills, the participant can achieve experiences of completely forgetting oneself [36].

There is a parallel between participating in the SUP activity and engaging in crafts, needlework, knitting, crocheting, weaving, etc., according to the principles of craft psychology, as explained by Anne Kirketerp [37], who also, among others, refers to the theories of Csikszentmihalyi. During the COVID-19 pandemic, a boom in regards to the participation in different forms of crafts was noted, and this boom has increased ever since. According to craft psychology, concentration is required while working on a craft or an activity that requires the use of the hands, and this process is very beneficial for the brain [37]. In a study with 200 respondents, Kirketerp found that this process brought about experiences of flow, peace, being in the present, forgetting oneself, and release from rumination [54,55]. The participants reported that they experienced presence and satisfaction by being completely engrossed in their craft work in a way that they lost the sense of time and saw the world in a new and more constructive perspective. We argue that these benefits of flow, peace, being in the present time, forgetting oneself, and release from rumination can not only be achieved by participating in craft work, but also from other meaningful and motivating activities demanding concentration, e.g., the SUP activities included in our study. Additionally, Kirketerp also emphasizes that small repetitive movements, as when parents rock their child in their arms, calm down the nervous system [37]. In our study, the rocking movements of the waves also possibly contributed to the participants feeling calm and peace, being in the present time, forgetting oneself, and experiencing released from rumination.

Finally, the perspectives of Merleau-Ponty also deliver a possible explanation for why the participants (bodily) activities demanding fully concentration paved the way for being in the present and keeping calm, without rumination. According to these perspectives, the body is regarded as an anchor to the now because the individual is at one with the body [39]. Our body is the closest we can come to the present [56], and the body is the present’s place; from its position in the present, the body can (re)establish contact with the mind, bringing the mind back to the present [57].

According to Killingsworth and Gilbert, spending more time in the present moment redirects our attention away from mind wandering and distraction towards what is happening right here and right now, in the present moment, thus leading us to greater happiness and well-being in general [49].

The participants did not experience such a break from rumination in any other activities.

The participants emphasized that having full focus on the difficult and challenging SUP activity gave the mind a break from rumination and a break from diagnoses, both in the present moment and beyond, and it was followed by a feeling of freedom in their daily lives. The participants experienced not only mastery, but a more comprehensive feeling of success in their lives which was not limited to just the specific moment.

These results help to highlight the lack of and the need for studies exploring the long-term influence of participating in blue space interventions on mental health [2,3,50].

Supporting the long-term sustainability of the mental health benefits, the theories of Csikszentmihalyi emphasize that optimal experiences will gradually, over time, build up a feeling of management and control in regards to creating one’s own life [36], and Pasanen found that physical activity outdoors in nature provided persistent well-being, which was even more persistent than that provided by indoor physical activity [51].

Finally, Fredrickson’s broaden-and-build theory also points out the sustainability and persistent potential for positive experiences and emotions to build personal resources and fuel individual differences in regards to resilience and the power of self-confidence, facilitating personal growth, and over time, building psychological resilience and increasing enduring coping resources [32].

All this underlines the long-term positive influence of SUP on mental health.

### 4.4. Theme 4: Connectedness and Being Part of Something Bigger

#### 4.4.1. Theme 4a: Connectedness to Nature, Specifically Blue Nature

Connectedness meant a lot to the participants. Connection with the group of peers, as well as connection with oneself and with nature, were important.

In a nature-based mindfulness study in Denmark, Djernis et al. also found that the sense of connection with oneself, others, and nature, and the sense of being away from everyday life and immersed in nature, were the most emphasized qualities supporting the capacity for self-regulation [58]. In a large study, with 524 adults participating in a nature project in natural areas in Italy, Berto et al. found that people who experienced connectedness to nature received more of the beneficial restorative effects from nature exposure [59]. Also supporting our results, another study by Sidenius et al. [60] found that connections with the group, with oneself, and with nature are interrelated. A total of 42 people dealing with stress conditions participated in a 10-week nature project, and they found that the mindful activities in a garden provided a basis for becoming more aware of themselves as “human wholes” [60].

The participants in our study had opened their eyes to both green and blue nature, and most of them even emphasized that being in, on, or by the water provided a distinct and unique perspective on spending time in nature, in contrast to being on land. Nature had become a greater part of their lives, improving their mental health and overall well-being with feelings of calmness and a sense that everything would be all right, as well as feelings of positivity, grounding, healing, free space, and a sense of freedom. As one of the participants said: “Those days where I’m really struggling with my disease, I go out and feel the nature as a calm and peaceful place and I just listen to the birds” (P4).

The participants had become more sensitive towards nature. As another participant said: “Now I’m like discovering nature every time I’m out for a walk then I see something new and interesting” (P2), and nature offered a feeling of a being a part of something bigger, being a part of a kind, embracing, floating, and calming natural movement saying, “Don’t worry, it will be all right” (P2). Nature also offered an all-embracing feeling of grounding, of “knowing where I am in the world, smelling the rain and the forest floor, making disturbing thoughts fly away and disappear” (P4).

It seemed that nature created a sort of connection to the participants’ childhood and their upbringing. It brought about memories of walking along the seaside as a kid, of the sounds of waves lapping and the smell of the water, together bringing calmness and a free space for positive thinking. The fact that memories brought the mind back to a safe and secure setting, as a basis for developing positive thinking and feelings, is supported by the theories regarding biophilia. The biophilia theories concern human connectedness to nature, and the term philia refers to a safe and secure setting as a basis for developing positive feelings [40,41]. Our connection to nature is genetic, and impressions through the senses from nature can foster an emotional and physiological response [41]. Biophilia has its roots in two Greek words, bio means life, and philia means friendship, often also translated as love [61]. Biophilia was first introduced in 1973 in by Erich Fromm, a German psychoanalyst, and in 1984, it was taken up by Edward O. Wilson, an American entomologist, who, in his book *Biophilia*, described biophilia as the deep relationship that human beings have with nature [40,61].

There is increasing evidence that interactions and connectedness with nature benefit people’s well-being, increase life satisfaction, improve attention restoration and stress recovery, and have a significant effect on increasing positive and decreasing negative emotion [52,53,62,63,64]. In a time with a great disconnect between humans and the rest of the natural world, it is more important than ever, to humans and to nature, that we support positive biophilic tendencies through direct and active engagement. Nature connectedness refers to how we relate to and experience nature, and a close connection with nature can pave the way for better mental health, and furthermore, for growing a mindset for taking active care of nature [52,53,65].

The evidence is clear that physical activity itself has a positive impact on mental health [66,67,68]. It is also evident that physical activity outdoors in nature reduces negative emotions and improves mental health [51,69,70], and Pasanen et al. even found that physical activity outdoors in nature seems to motivate more persistent well-being than physical activity indoors [51].

#### 4.4.2. Theme 4b: Connectedness to the Group of Peers

Being together with like-minded people in a group of peers also created an essential and valuable feeling of being a part of something, i.e., something bigger than what could be created alone. It created a feeling of being a part of a team, a feeling of having colleagues, and a sense of belonging. The participant did not have to answer for things and explain everything; there was an underlying understanding for and acceptance of each other, leading to increased self-confidence and a greater acceptance of oneself and of one’s own life situation. The sense of belonging was emphasized as a lifestyle-changing component, paving the way for either continuing to participate in SUP or other activities, and for an evolving social life and new friendships. This ballast had great life-changing potential. However, two participants (P1, P2) mentioned that for them, the sense of belonging changed over time along with changes in group dynamics when new participants arrived and joined the group. For them, this change led to a decrease in their sense of belonging, and they felt a change in the structure of Freedom on Water. This indicates another aspect of blue space interventions, which focuses on the structure and design of the interventions instead of the outcomes. This aspect could be interesting to explore in another investigation, as a review also highlights the need for a more detailed evaluation of the design and delivery of blue space interventions [1].

### 4.5. Theme 5: A Life Changing Journey; a Mindset Shift

In general, the participants expressed that Freedom on Water had changed their lives and had paved the way for new opportunities. The participants had acquired more self-confidence, self-respect, and additional energy, and moreover, their mindsets had become more out-turned towards society.

As P2 expressed: “I couldn’t have done without it. Otherwise, I don’t think I would be here today, if it had not been for Freedom on Water, I simply don’t think so (…) It was almost life-saving for me. At the time I started, I couldn’t do anything”. The participant went on to explain that by learning to shift her focus and be present in her body, she now, three and a half years later, believed she had an amazing life, despite her ongoing limitations. She had come to accept this reality instead of viewing it negatively. And as P6 expressed: “So, I was also feeling insecure about the fact that I was going to be together with other people in that way and with so many people—well, I think that has changed now. So, I’ve become happier, and I dare to call around so we can go on the water together (…)”.

The participants had developed an outward mindset, and in the perspective of Gert Biesta [42], they had become more world-centered, turning their attention outward to the world. This initially began with feeling a colleagueship within the group of peers, and later, they began making new friendships, participating in different contexts, both with the “regular” club members and in new groups, initiating new activities, alone or in groups, and some even took leadership roles. Overall, the participants had embraced a range of new activities, spanning from social gatherings to water gymnastics, discovering novel ways to appreciate nature and specifically, blue nature. According to Biesta, we can meet the world with two different approaches [42]. We can meet the world with the purpose of understanding it and with an attitude that the world is for the humans; the humans are the center. The other approach to the world is quite different. It means meeting the world with the attitude that the world is coming to us, the world gives itself to us, the world surprises us, the world controls us, and it call us to experience it. In this approach, we are not the center as humans; we are not controlling the world as humans. This means that I can relax and let go, I can leave the world and nature in peace and be a part of it, taking care of it because it is the center.

A minor 10-month follow-up study on nature-based surf therapy also revealed that a mindset shift was initiated for the six participants, leading them to adopt well-being-promoting long-term behavior changes [48].

Carol S. Dweck distinguishes between two different mindsets, growth and fixed [71]. In a fixed mindset, everything is about the outcome, and in a growth mindset, you value what you are doing, regardless of the outcome, and you tackle problems and challenges as you meet them. In other words, you are in the present moment, experiencing what you are doing right now (e.g., the activity; the process). In this perspective the participants developed a growth mindset when they stepped out of their comfort zones and discovered that participating in challenging activities fostered growth and learning. As described above when P1, afraid of falling into the water, fell into the water, it turned out to be an experience of success because she managed to get up again so quickly that nobody noticed, she got the feeling of, “Wow, I did it well, yes!”. According to Dweck, changing our beliefs can have a powerful impact, and the growth mindset creates a powerful passion for learning [71]. This theory supports the potential long-term lasting mindset shift.

Obviously, the instructors were capable of creating a safe platform for fostering a mindset-shift to a growth mindset.

### 4.6. Limitations and Strengths

#### 4.6.1. The Importance of This Study

This study employs a small sample size, consisting of six participants and two instructors, which limits its generalizability and calls attention to the need for more research exploring the long-term influence of blue space interventions on well-being and mental health. Nevertheless, our findings are important because they meet the need for long-term research in the area of blue space interventions. No other research, apart from our study, has considered the long-term effects of such interventions for more than one year.

Until now, the long-term effects have only been considered for shorter periods spanning a few weeks and months to one year [2,3,43,44,45,46,47,48]. A systematic review emphasized that the short-term benefits of blue space interventions were well reported; however, very few studies considered the long-term effects, and none for a period of more than one year [2]. Over half of the studies used a pre–post design, and of these, only three assessed the participant’s experiences during the intervention. Only five studies considered the long-term effect, and it was only considered for three months to one year [2]. A scoping review of the qualitative and quantitative research evidence for surf therapy also emphasizes the literature gap concerning both the immediate and long-term effects of surf therapy interventions [3]. This review found that several studies included a follow-up period from between two to three weeks, but none of the studies included a follow-up for a time-period beyond one year. Together, these studies underpin the importance of our results regarding the long-term influence of blue space interventions.

When conducting long-term research, it is reasonable to consider the fact the participants have experienced other things in their lives which could also have had an influence on their mental health. Nevertheless, based on the experiences of the participants in Freedom on Water, the intervention evidently has had a positive impact on the participants’ mental health and daily lives.

#### 4.6.2. Methods

Intermediate evaluations were not a part of our study and could have strengthened the results.

#### 4.6.3. Increased Awareness Verbalized Tacit Knowledge

Especially in the beginning, the participants lacked words that could fully describe the benefits they experienced from being in, on, or by the water. It is well known that knowledge obtained through (bodily) experiences and motor skills is typically difficult to verbalize spontaneously and therefore, is often described as tacit knowledge or implicit knowledge [72]. By increasing attention and awareness, over time, subjects may be able to reveal some of this tacit knowledge, and this most likely is what happened in our study. Although it was still difficult to express in words, the participants were aware of their experiences and the benefits they received, and they were able to verbalize them in this follow-up study. Also, participating in these two studies contributed to increased awareness and the ability to articulate the experiences. These results support the validity of this study.

## 5. Conclusions

Our two-and-a-half-year follow-up study showed that participating in a group-based Freedom on Water intervention with stand-up paddling in a group of peers (among people with mental disorders), guided by two instructors, had a long-term, life-changing impact on the participants’ well-being, mental and social health, and lives. It enabled the participants to change their lives and their mindsets, with more confidence in themselves and their own abilities, and with more of an outward focus on society. The participants learned that stepping out of their comfort zones and balancing between challenges and skills gave them gateway experiences of success and broadened their horizons. Together with the group of peers, they felt that they were part of something bigger. They built up a colleagueship within this group, they made new friendships, and they began participating in new and different contexts, some even taking leadership roles. In other words, they became more world-centered. They experienced breaks from their rumination lasting longer than the SUP sessions, giving them additional energy in their daily lives, and they achieved a feeling of being much more than merely their diagnosis. The participants opened their eyes to and became more sensitive towards both green and blue nature, and specifically, blue nature now meant a lot to them. Nature had become a greater part of their lives, improving their mental health and overall well-being with feelings of calmness, safety, and that “everything will be all right”, as well as feelings of positivity, grounding, healing, free space, and a sense of freedom. On the whole, it had been a life changing journey which had fostered a mindset shift and opened their minds to be more outward focused and world-centered, with more confidence in themselves and their own abilities and with additional energy and initiative for participating in other things, beyond Freedom on Water—and they were still participating in these “other things”. Our findings are important because they fulfill the need for long-term research in the area of blue space interventions. No other research, apart from our study, has considered the long-term effects of such interventions for more than one year.

## Figures and Tables

**Table 1:** Step Two.

Theme: Nature and Water	Empirical Data
Nature and water	“I specifically strive to see some water, like if it is possible to go for a walk along the water then I just feel really good. Then I can imagine myself being out there where nobody can reach me, and I can just be in the moment”.

**Table 2:** Step Three.

Theme: Nature and Water	Empirical Data
Sub-group: changes in perceptions of nature	“Water didn’t really mean anything to me, it wasn’t something special, neither was nature, but now I appreciate it more. I have a desire to go out, like now I can see that nature has more opportunities, that you can go out and enjoy it in several ways”.

## Data Availability

Data can be obtained from ebo@via.dk.

## Appendix A

### Appendix A.1. Interview Guide for Participants (Primary Participants)

Briefing

- Presentation
- Setting of the interview
- Guidelines, declaration of consent, and anonymization
- Possible questions

Freedom on Water

- How long have you been participating/or did you participate in Freedom on Water?
- Why have you chosen to continue to participate?/How is your relationship with SUP since you are no longer participating in Freedom on Water?

Change within experiences

- If you think back to the first time you participated in Freedom on Water until now, how would you describe that journey?
- What experiences including water/SUP did you have before you started participating in Freedom on Water?
- What experiences do you have now with water/SUP besides Freedom on Water? Or after you have stopped participating?

Significance of SUP/water

- What does SUP/water mean to you?
- How has this meaning possibly changed from the first time you were on the water until now?
- How have these experiences or possible changes affected you?

Experiences

- How would you describe your relationship towards nature/water? If there has been a change in this since Freedom on Water started, can you describe how it has changed?
- How would you describe your motivation to get on the water? Do you think you would get on the water on your own if it was not through Freedom on Water?

Debriefing

- The participant’s own additions
- Thank you

### Appendix A.2. Interview Guide for Instructors (Secondary Participants)

Briefing

- Presentation
- Setting of the interview
- Guidelines, declaration of consent, and anonymization
- Possible questions

Experiences on the water

- For how long have you been an instructor with Freedom on Water?
- If you think back to the first session you held as an instructor with Freedom on Water until now, how would you describe that journey? What possibly changes have you experienced?
- How do you experience having the participants with you on the water?

Significance of experiences

- Why do you think that the participants have chosen to continue with SUP/Freedom on Water?
- How have you experienced/observed the impact that Freedom on Water has had on the participants in the moment on the water vs. in the long run?
- What do you think is necessary for the participants to achieve changes?

The participants’ development through Freedom on Water

- How have you experienced the well-being of the participants (from the beginning until now)?
- What changes have you experienced/observed among the participants (from the beginning until now)?
- How do you consider the participants’ retention/motivation to continue SUP (also, if Freedom on Water was to end)?

Debriefing

- The participant’s own additions
- Thank you
